# Summary of best evidence on health education for pediatric urinary tract infections

**DOI:** 10.3389/fpubh.2026.1831040

**Published:** 2026-05-28

**Authors:** Xuqiong Tan, Jianrong Liao, Fengbi Jiang, Yun Li, Ping Zhou

**Affiliations:** 1 Department of Pediatric Nephrology and Rheumatology, Sichuan Provincial Maternity and Child Health Care Hospital, Sichuan Provincial Women’s and Children’s Hospital, Sichuan Clinical Research Center for Pediatric Nephrology, Chengdu, China; 2 School of Nursing, Chengdu Medical College, Chengdu, Sichuan, China; 3 Sichuan Provincial Women’s and Children’s Hospital / The Affiliated Women’s and Children’s Hospital of Chengdu Medical College, Chengdu, China

**Keywords:** children, evidence summary, health education, prevention, urinary tract infection

## Abstract

**Background:**

Pediatric urinary tract infections (UTIs) significantly affect children’s health and quality of life. Health education plays a key role in improving parental health literacy, treatment adherence, and recurrence prevention. This study aimed to systematically synthesize the best available evidence on health education for the prevention and management of pediatric UTIs to serve as a reference for clinical practice.

**Methods:**

Following the “6S” evidence resource model, an evidence search was conducted in a top-down manner across the following sources: BMJ Best Practice, UpToDate, National Institute for Health and Care Excellence (NICE), Guidelines International Network (GIN), National Guideline Clearinghouse (NGC), Registered Nurses’ Association of Ontario (RNAO), Canadian Medical Association (CMA), New Zealand Ministry of Health Guidance Library, MedLive, American Academy of Pediatrics (AAP), European Association of Urology (EAU), Cochrane Library, EMbase, Ovid MEDLINE, PubMed, Web of Science, CINAHL, China National Knowledge Infrastructure (CNKI), Wanfang Data, VIP, and SinoMed. The search period covered from database inception to October 31, 2025. Two reviewers independently screened and evaluated the retrieved literature. Evidence was extracted and summarized according to the JBI evidence grading and recommendation system.

**Results:**

A total of 14 publications were finally included: 4 clinical decisions, 5 guidelines, 1 evidence summary, 3 expert consensuses, and 1 systematic review. Through evidence synthesis and integration, 42 best-evidence statements were developed across eight domains: disease awareness, symptom recognition, diagnosis and evaluation, urine specimen collection, imaging examinations, treatment and medication, recurrence prevention, and follow-up instructions.

**Conclusion:**

This best-evidence summary comprehensively synthesizes evidence-based health education for pediatric urinary tract infections. The rigorous methodology and broad content coverage provide valuable scientific guidance for healthcare professionals involved in UTI health education.

## 1 Introduction

Urinary tract infection (UTI) is one of the most common infectious diseases in children, characterized by a high incidence and a propensity for recurrence (1). Clinical manifestations vary by age, with symptoms often atypical in young infants and toddlers, leading to frequent missed or incorrect diagnoses (2). Although modern diagnostic and therapeutic approaches have reduced mortality rates to nearly zero, UTIs remain a significant cause of renal scarring, urinary stones, hypertension, progressive renal damage, and renal failure in children (3). Since daily care for affected children is primarily provided by non-professional caregivers such as parents, UTI prevention depends heavily on sustained home management. Timely identification of risk factors and implementation of preventive measures are crucial to avoiding disease onset and recurrence. Health education plays a pivotal role in disease prevention and health promotion; however, current educational content for pediatric UTIs is limited and lacks systematic, comprehensive, evidence-based guidance. Additionally, parents demonstrate a significant demand for information regarding the prevention, diagnosis, treatment, and prognosis of pediatric UTIs (4).

Therefore, this study adopts a disease lifecycle management perspective, integrating the background, needs, and preferences of affected children. Using evidence-based methods, it synthesizes the best available evidence to provide comprehensive health education throughout the pediatric UTI cycle. To our knowledge, no prior evidence summary has systematically collected and graded pediatric UTI health education evidence across the full disease continuum from 14 top-tier sources. This study offers a caregiver-oriented, actionable framework that translates clinical guidelines into practical health education messages. It provides healthcare professionals with a reference for developing holistic health education strategies.

## 2 Method

### 2.1 Search strategy

According to the ‘6S’ evidence resource model, evidence retrieval is searched from the top-down. The databases searched included: BMJ Best Practice, Up To Date, National Institute for Health and Care Excellence (NICE), Guidelines International Network (GIN), National Guideline Clearinghouse (NGC), Registered Nurses’Association of Ontario (RNAO), Canadian Medical Association (CMA), New Zealand Ministry of Health Guidance Library (NZ MOH Guidance Library), MedLive, American Academy of Pediatrics (AAP), European Association of Urology (EAU), Cochrane Library, EMbase, Ovid MEDLINE, PubMed, Web of Science, CINAHL, China Knowledge Resource Integrated Database (CNKI), Wanfang, VIP, and SinoMed.

The search terms were created based on the combination of Medical Subject Headings andfree terms, and search encompassed clinical decisions, guidelines, evidence summaries, expertconsensus statements, and systematic reviews. The search terms are “Child/children/childhood/pediatric/toddlers/infants/pediatric patients/young children”; “urinary system infection/urinary infection*/urinary tract infection/urine tract infection/genitourinary tract infection/UTI”; “health education/healthy education/education/health care education/Community Health Education/management/nursing”; “guideline/clinical practice guideline*/Best practice/Meta-Analysis/Systematic Review/consensus.”The search strategy for PubMed is presented as follows:((((“Urinary Tract Infections”[Mesh]) OR (“urinary system infection”[Title/Abstract] OR “urinary infection*”[Title/Abstract] OR “urine tract infection”[Title/Abstract] OR “genitourinary tract infection”[Title/Abstract] OR “UTI”[Title/Abstract])) AND ((“Health Education”[Mesh]) OR (“healthy education”[Title/Abstract] OR “education”[Title/Abstract] OR “health care education”[Title/Abstract] OR “Community Health Education”[Title/Abstract] OR “management”[Title/Abstract] OR “nursing”[Title/Abstract]))) AND ((“Child”[Mesh]) OR (“children”[Title/Abstract] OR “childhood”[Title/Abstract] OR “pediatric”[Title/Abstract] OR “toddlers”[Title/Abstract] OR “infants”[Title/Abstract] OR “pediatric patients”[Title/Abstract] OR “young children”[Title/Abstract]))) AND ((“Guidelines as Topic”[Mesh]) OR (“guideline”[Title/Abstract] OR “clinical practice guideline*”[Title/Abstract] OR “Best practice”[Title/Abstract] OR “Systematic Review”[Title/Abstract] OR “consensus”[Title/Abstract])). The search spanned from database inception to October 31, 2025.

### 2.2 Literature inclusion and exclusion criteria

*Inclusion criteria*: (1) Clinical decision, guidelines, evidence summaries, systematic reviews, meta-analyses, or expert consensus on health education content for pediatric UTIs; (2) the latest version of guidelines; (3) draft or trial versions when formal guidelines are unavailable; (4) published in Chinese or English.

*Exclusion criteria*: (1) full text unavailable; (2) Guideline interpretations, direct translations, or duplicate publications.

### 2.3 Literature quality evaluation criteria

The guidelines were appraised using the Appraisal of Guidelines for Research and Evaluation II (AGREE II) framework (5), which includes 23 items across six domains, each scored 1–7. Recommendation grades (A, B, C) were determined based on domain-specific standardized percentages (calculated as: (obtained score − minimum)/(maximum − minimum) × 100%). Evidence summaries were evaluated using the CASE tool (6). Expert consensus and systematic reviews were assessed using JBI critical appraisal checklists (7). High-grade Clinical decision-making were directly referenced (8). Three researchers independently appraised guidelines; two researchers appraised other document types. Disagreements were resolved through discussion or consultation with evidence-based nursing specialists.

### 2.4 Evidence extraction and synthesis

Two reviewers independently screened the retrieved literature against predefined inclusion and exclusion criteria. Disagreements were resolved through discussion until consensus was reached or by consulting a third reviewer. Evidence synthesis followed three principles: (1) when content was consistent, selecting concise, clear, and professionally appropriate evidence; (2) when content was complementary, merging evidence logically; and (3) when content conflicted, prioritizing evidence-based studies, high-quality evidence, and the most recent authoritative sources. Evidence was graded according to the 2014 JBI Evidence Grading System (Levels 1–5) (9). The strength of recommendations (A: strong, B: weak) was determined based on the JBI FAME structure (Feasibility, Appropriateness, Meaningfulness, Effectiveness).

## 3 Results

### 3.1 Literature screening results

**Figure 1:**
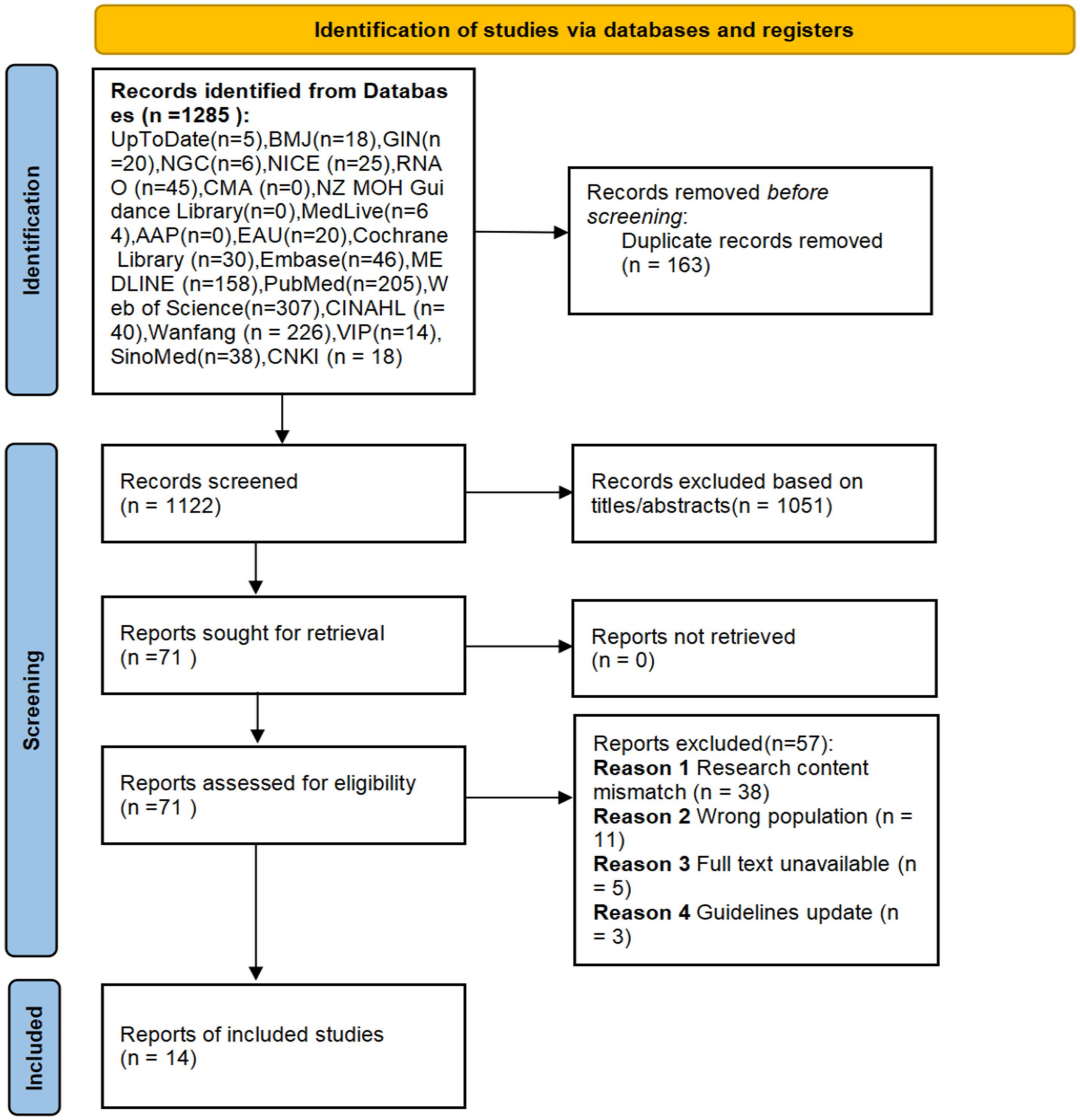
PRISMA flow diagram of the literature search and selection process for the best evidence summary.

A total of 1,285 publications were retrieved, of which 14 met the inclusion criteria after screening (3, 10–22). The study selection process is presented in Figure 1, and the general information of the included literature is shown in Table 1.

**Table 1:** General information of the included literature (*n* = 14).

Included literature	Literature sources	Year	Type of evidence	Topic of the literature
Nader et al. (10)	UpToDate	2025	Clinical decision-making	Long-term treatment and prevention of urinary tract infections in children
Duncan et al. (11)	UpToDate	2025	Clinical decision-making	Care and complications of the uncircumcised penis in infants and children
Kenneth et al. (12)	UpToDate	2024	Clinical decision-making	Management of bladder dysfunction in children
Lalit et al. (13)	UpToDate	2024	Clinical decision-making	Urine collection techniques in infants and children with suspected urinary tract infection
NICE guideline (14)	NICE	2022	Guideline	Diagnosis and management of urinary tract infections in children under 16 years of age
Yang et al. (15)	MedLive	2021	Guideline	Management of urinary tract infections and recurrence in children
Hari et al. (16)	MedLive	2024	Guideline	Management of urinary tract infection and primary vesicoureteric reflux
Hoen et al. (17)	MedLive	2021	Guideline	Management and diagnostic evaluation of pediatric urinary tract infections
Ammenti et al. (18)	MedLive	2019	Guideline	Diagnosis, treatment, and follow-up recommendations for first-time fever-associate Urinary tract infections in young children
Zhang Caifeng et al. (19)	CNKI	2020	Evidence summary	Summary of non-invasive collection of infant and toddler urine samples
Subspecialty Group of Neonatology, Society of Pediatrics, Chinese Medical Association (20)	CNKI	2025	Expert consensus	Management of neonatal urinary tract infections
Buettcher et al. (3)	MedLive	2020	Expert consensus	Current recommendations for the management of urinary tract infections in children
Autore et al. (21)	PubMed	2022	Expert consensus	Management of pediatric urinary tract infections
Uwaezuoke et al. (22)	Ovid MEDLINE	2019	Systematic review	Prevalence and risk factors for urinary tract infections in malnourished children

### 3.2 Literature quality assessment results

This study included five guidelines (14–18), Quality assessment results are shown in Table 2. Three expert consensuses (3, 20, 21) met all six criteria and were included. One systematic review (22) met all 11 criteria and was included. One evidence summary (19) met all 10 criteria and was included. Additionally, four Clinical Decision-Making (10–13) were directly cited as high-level evidence.

**Table 2:** Methodological quality evaluation results of the guidelines (*n* = 5).

Included guidelines	Percentage of field standardisation %						≥60% field number (*n*)	≥30% field number (*n*)	Recommendation level
	Scope and purpose	Participants	Rigour of development	Clarity	Applicability	Independence			
NICE guideline (14)	100	92.56	91.67	100	80.56	100	6	6	A
Yang et al. (15)	98.15	74.07	51.39	87.04	47.22	88.88	4	6	B
Hari et al. (16)	100	90.07	87.5	96.3	59.72	94.44	5	6	B
Hoen et al. (17)	100	83.33	85.42	98.15	68.06	100	6	6	A
Ammenti et al. (18)	100	68.52	75	98.15	23.26	100	5	5	B

Standardisation percentage of each field = (obtained score − minimum possible score)/(maximum possible score − minimum possible score) × 100%; Recommendation level: if the standardised percentage of six fields is >60%, it is highly recommended (level A); if >3 areas have a standardised percentage >30% and <60% are recommended (level B); if there are ≥3 areas with a standardised percentage <30%, it is not recommended (level C).

### 3.3 Summary and description of evidence

The evidence was extracted from the final literature, and the evidence was evaluated by JBI grading of evidence and recommendation system. Through the induction and integration of the evidence, the evidence was finally summarized from eight aspects: Disease awareness, Symptom recognition, Diagnosis and evaluation, Urine Specimen Collection, imaging examinations, therapeutic medication, recurrence prevention and Follow-up Instructions, and 42 best evidences were formed, as shown in Table 3.

**Table 3:** Summary of the best evidence for health education on urinary tract infections in children.

Theme	Summaried evidence for health education	Clinical application	Evidence level	Recommendation grade
Disease awareness	1. All children with unexplained fever should be evaluated for the possibility of urinary tract infection (3, 18).	Teach caregivers that unexplained fever may be the only sign of UTI and should prompt timely medical assessment rather than home observation alone.	1	A
	2. UTI risk factors: female infants, urinary tract anomalies, preterm infants, prolonged hospitalization, invasive procedures, phimosis, constipation, bladder-bowel dysfunction, and inadequate daily fluid intake (3, 20, 21).	Screen for modifiable risk factors during education and provide individualized counselling on fluid intake, bowel habits, phimosis care, and voiding behavior.	2	A
	3. *Escherichia coli* is the most common pathogen in pediatric urinary tract infections, accounting for over 70% of cases, followed by Klebsiella spp., Enterobacter spp., and Proteus spp. (21).	Use simple language to explain common pathogens and the need for culture-guided therapy, helping caregivers understand why antibiotics may be adjusted after results return.	3	A
	4. Hospitalization is recommended for severe infections (e.g., sepsis), dehydration, vomiting, difficulty managing home medications, or persistent fever lasting more than 3 days despite standardized treatment (18).	Provide clear warning signs requiring hospital care, including dehydration, vomiting, sepsis-like symptoms, persistent fever, and inability to take medication at home.	5	A
	5. Urinary tract infections should be classified based on the site of infection (upper or lower urinary tract), frequency of occurrence (first episode or recurrent), severity (uncomplicated or complicated), and the presence of complicating factors (e.g., urinary tract anomalies). This classification helps assess the risk of renal injury in pediatric patients (15, 21).	Explain UTI classification in relation to recurrence risk and possible kidney injury so families understand why assessment and follow-up differ across children.	2	B
	6. Following a first urinary tract infection, approximately 30 to 50% of children will experience recurrence (21).	Inform families that recurrence is common after a first UTI and provide a written prevention and follow-up plan before discharge.	5	B
Symptom recognition	7. Typical symptoms include painful or difficult urination, frequent urination, sudden bedwetting, foul-smelling urine, cloudy urine, or visible blood in the urine. These symptoms may be accompanied by fever, chills, abdominal pain, or tenderness in the lower back upon percussion (14).	Provide an age-appropriate symptom checklist covering urinary symptoms and systemic signs, and instruct caregivers when to seek care.	5	A
	8. Atypical symptoms, especially in infants, primarily include systemic signs such as fever (or hypothermia), lethargy, feeding difficulties, vomiting, diarrhea, jaundice, and failure to thrive, without specific urinary tract manifestations (14).	For infants, emphasize non-specific symptoms such as fever/hypothermia, poor feeding, vomiting, diarrhoea, jaundice, lethargy, and poor growth.	5	A
	9. Special Note: In infants under 3 months of age, the presence or absence of fever does not correlate with the severity of a UTI (21).	Tell caregivers of infants under 3 months that absence of fever does not exclude severe UTI; encourage early consultation when general condition changes.	5	A
Diagnosis and evaluation	10. Diagnostic criteria: Urine culture is the gold standard for diagnosis (18).	Emphasize that urine culture confirms diagnosis; whenever possible, collect urine before antibiotics and avoid contamination.	1	A
	11. Post-Diagnosis Evaluation: ① Children who have mastered toilet training but experience recurrent fevers and recurrent UTIs should be evaluated for bladder and bowel dysfunction. ② For a first febrile UTI caused by pathogens other than *Escherichia coli*, an evaluation for vesicoureteral reflux is recommended. ③ Children over 1 year of age with febrile UTIs caused by *Escherichia coli* should be evaluated for vesicoureteral reflux after a second episode (17).	Educate families of recurrent or febrile UTI about the need for BBD or VUR evaluation and explain why repeated episodes may require further testing.	2	A
	12. Preliminary Screening: For children with unexplained fever (temperature >38 °C) or presenting symptoms suggestive of a urinary tract infection (UTI), an initial assessment should be conducted based on medical history, symptom and sign observation, and physical examination. This should be supplemented with rapid urine test strip screening for auxiliary evaluation (17, 21).	Guide caregivers to cooperate with history-taking, symptom observation, physical examination, and rapid urine testing when UTI is suspected.	3	A
	13. Diagnostic Basis: Positive urine tests and urine cultures are essential diagnostic criteria for urinary tract infections. Urinalysis strips primarily assess two indicators: leukocyte esterase and nitrite. A positive result for both markers indicates high diagnostic accuracy for UTIs. A positive nitrite result alone demonstrates high specificity but carries a risk of false negatives, while a positive leukocyte esterase result alone shows high sensitivity but may produce false positives. A negative result for both markers generally rules out a urinary tract infection (3, 15, 18, 20, 21).	Explain the meaning and limitations of leukocyte esterase, nitrite, urinalysis, and culture results to reduce misunderstanding and improve cooperation.	3	B
Urine specimen collection	14. Specimen Collector: Urine specimens should be collected by trained medical personnel (19).	Specify that collection should be performed or supervised by trained staff to improve sample quality and reduce false-positive results.	1	A
	15. Cleaning and Disinfection: ① Operators must first wash their hands with soap and water or wipe them with disinfectant wipes; ② Thoroughly clean the infant’s genital area with warm water and soap (paying particular attention to female infants and male infants with phimosis), then dry the area with sterile gauze (19).	Demonstrate hand hygiene and genital cleaning steps before collection; use teach-back to confirm caregiver understanding.	1	A
	16. Specimen Collection Methods for different populations: ① Infants and toddlers who are not yet toilet-trained: Preferably obtain specimens via catheterization or suprapubic bladder puncture. If non-invasive methods are necessary, use urine collection bags; strictly avoid using plastic bags, cotton balls, gauze, or sanitary pads. ② FeverishFebrile pediatric patients in poor clinical condition: Specimens should be collected through catheterization or suprapubic bladder puncture. ③ Toilet-trained children: Clean-catch midstream urine may be used for screening; however, definitive diagnosis requires culture of specimens obtained via catheterization or suprapubic puncture (3, 14, 15, 17).	Teach age-specific collection methods and clearly state which non-standard materials should not be used because of contamination risk.	2	A
	17. Timing for Collection: Before collecting infant or toddler urine samples, provide an appropriate amount of food based on the child’s age and individual needs (feeding duration ≤20 min; extend as necessary for those with feeding difficulties). Collection should begin 20–25 min after feeding (18, 19).	For infants/toddlers, coordinate feeding and collection timing; explain the rationale to parents to improve collection success.	2	A
	18. Collection Assistance Techniques: For newborns and infants under 6 months of age, bladder stimulation techniques may be used to collect urine specimens. For infants aged 6 months and older, as well as for children, urine specimens should be collected using a urine collection bag (19).	Use bladder stimulation or collection bags only when appropriate; provide demonstration and supervision for caregivers when non-invasive methods are used.	2	A
	19. Specimen Submission: Specimens must be submitted promptly after collection. If immediate submission is not possible, store specimens in a refrigerator at 2–8 °C or preserve them with a boric acid solution. Cultivation remains viable for up to 24 h (13, 14).	Give written instructions on rapid submission and cold storage when delay is unavoidable; document time of collection and submission.	1	B
Imaging examinations	20. Renal and Bladder Ultrasound (RBUS) is indicated for all pediatric patients with urinary tract infections to screen for structural abnormalities of the urinary system (21).	Explain the purpose of RBUS as screening for urinary tract structural abnormalities, not as a routine “severity test” for every symptom.	5	A
	21. Voiding cystourethrography (VCUG) is the gold-standard method for diagnosing vesicoureteral reflux (VUR). Indications for VCUG include: ① first febrile urinary tract infection caused by non-*Escherichia coli* bacteria; ② ultrasound findings of severe hydronephrosis, ureteral dilation, renal dysplasia, or a thickened bladder wall; ③ all second febrile urinary tract infections; ④ prior to planned anti-reflux surgery (21).	Explain VCUG indications and procedure preparation for selected children; emphasize that it is not required for every UTI episode.	5	A
	22. Timing for RBUS examination: ① Routine timing: 2–4 weeks after the first febrile urinary tract infection; ② Urgent timing: Infants with febrile urinary tract infections require RBUS examination within 24 h to rule out upper or lower urinary tract obstruction. ③ Acute phase: During a febrile UTI, RBUS is indicated only for complicated or atypical infections (e.g., sepsis, persistent fever after 72 h of adequate antibiotic therapy, elevated serum creatinine, oliguria, or infections caused by non-*E. coli* pathogens). Testing is not recommended during the acute phase otherwise (17, 18).	Clarify recommended timing for RBUS according to age, febrile status, atypical infection, and clinical severity to reduce unnecessary anxiety.	5	B
Therapeutic medication	23. Therapeutic Monitoring: Three to 5 days after the initial diagnosis and the start of empirical antibiotic therapy, reassess treatment efficacy by: ① reviewing urine culture results and adjusting medications based on antimicrobial susceptibility testing (e.g., narrowing the antibiotic spectrum); ② discontinuing empirical antimicrobial therapy and considering alternative diagnostic approaches if urine cultures show no significant bacterial growth ③.	Arrange reassessment within 3–5 days when indicated; teach caregivers to report persistent fever, worsening symptoms, or adverse drug reactions.	1	A
	24. Distinguishing the site of infection—whether it is an upper urinary tract infection (pyelonephritis) or a lower urinary tract infection (cystitis)—is crucial for selecting the appropriate treatment plan ③.	Explain that upper and lower UTIs may require different treatment plans, supporting adherence to individualized medical decisions.	1	A
	25. Treatment principles: Early diagnosis and early treatment. Empirical antibiotic therapy should be initiated immediately after obtaining a urine culture specimen (15, 20).	Educate caregivers that empirical antibiotics should start after urine culture collection when possible, and treatment should not be delayed in clinically indicated cases.	2	A
	26. Antibiotic selection: Before identifying the causative pathogen, empirical antibiotics may be selected based on common pathogens and local bacterial resistance patterns. Once the causative pathogen is identified, medication should be adjusted according to the results of antimicrobial susceptibility testing (3, 15, 18, 20).	Inform caregivers that antibiotic choice may change after susceptibility results; discourage self-changing or reusing previous antibiotics.	2	A
	27. Route of Administration Selection: ① Complicated UTIs (presence of sepsis, severe dehydration or vomiting, or compliance issues): Initiate intravenous therapy, transitioning to oral antibiotics when clinically appropriate. ② Uncomplicated UTIs (febrile children in stable clinical condition who can tolerate oral fluids and medications with good compliance): Oral therapy is preferred. ③ Neonates: Administer intravenous therapy throughout the entire course (18, 20).	Explain why intravenous or oral therapy is selected according to severity, age, vomiting/dehydration, sepsis risk, and adherence feasibility.	5	A
	28. Treatment Duration: ① Uncomplicated febrile UTI: Antibiotic course of at least 7–10 days; ② Complicated UTI: Course of at least 10–14 days; ③ Children over 3 months with infection confined to the lower urinary tract: Course may be shortened to 5 days; ④ Infants/neonates under 3 months: Intravenous treatment course of 10–14 days (15, 18, 20, 21).	Provide a treatment-duration card and stress completion of the full course even when symptoms improve.	5	B
Recurrence prevention	29. Antibiotic prophylaxis: Routine use for preventing recurrence after initial infection is not recommended (18). However, it may be considered for children with moderate-to-severe (Grade III-V) vesicoureteral reflux or recurrent febrile infections with risk of renal injury (15).	Explain that prophylactic antibiotics are not routinely required after a first UTI, but may be considered for selected high-risk children under clinician guidance.	1	A
	30. Bladder-bowel dysfunction (BBD) is a key factor in renal scar progression. Early identification and management of BBD are crucial for preventing recurrent urinary tract infections (15).	Screen for BBD and constipation and integrate bladder-bowel management into recurrence-prevention education.	2	A
	31. Other Prevention: In specific circumstances, dietary supplements may serve as alternative or adjunctive preventive measures (17).	Discuss supplements only as conditional adjuncts; emphasize that they should not replace evidence-based prevention, follow-up, or prescribed therapy.	2	A
	32. Foreskin Care: Instruct parents, caregivers, and growing boys on proper hygiene and care for the uncircumcised penis. During bathing, clean the penis with plain water or mild soap suitable for children of this age, using gentle motions similar to washing fingers, without forcibly retracting the foreskin. Additionally, change diapers frequently to prevent diaper rash and skin irritation (11).	Teach gentle foreskin hygiene without forceful retraction and provide diaper-skin care instructions for infants and young boys.	3	A
	33. Voiding Diary and Rewards: ① After initiating voiding behavior correction, maintain a daily voiding diary that records urination times and volumes, incontinence episodes, fluid intake, bowel movement times, and fecal incontinence episodes. ② Establish a positive reinforcement system to enhance compliance and boost self-confidence (12).	Use voiding diaries and positive reinforcement to improve adherence to behavioral interventions and enhance child participation.	3	A
	34. Nutritional Support: Children with malnutrition require enhanced nutritional support and should be included in routine urinary tract infection screening and treatment protocols to reduce the risk of recurrent infections (22).	For malnourished children, include nutrition assessment and support in the prevention plan and reinforce UTI screening when symptoms occur.	3	A
	35. Urinary Management: ① Establish Regular Urination: Educate family members and school-aged children about normal urination habits, develop personalized plans, and schedule timed voiding every 2–3 h during the day. Older children may use alarm clocks or timers as reminders. ② Standardize Voiding Behavior: Encourage urination before urgency peaks; adopt proper voiding posture; avoid straining positions such as forcefully crossing the legs or squatting while pressing the perineum with hands or heels (Vincent’s knee-bending posture). Implement the “double voiding method,” which involves sitting again immediately after the initial voiding to ensure complete bladder emptying without abdominal straining. ③. Ensure Adequate Facilities: Guarantee access to clean restrooms when needed to prevent delayed urination (10, 12, 14).	Develop a timed voiding plan, teach correct voiding posture and double voiding, and coordinate restroom access at school when needed.	5	A
	36. Surgical Intervention: For pediatric patients with recurrent febrile UTIs complicated by vesicoureteral reflux, surgical treatment may be considered (15, 16).	For recurrent febrile UTI with VUR, explain that surgical options may be discussed by specialists; avoid presenting surgery as routine prevention.	2	B
	37. Constipation Management: Administer mild laxatives promptly to children experiencing constipation. Long-term constipation in children can be effectively managed with standardized laxative therapy, which significantly reduces the recurrence of urinary tract infections. Additionally, address functional elimination syndrome and constipation in infants and children with a history of UTIs (14).	Educate caregivers to identify and treat constipation early; refer for standardized management when constipation persists.	3	B
	38. Fluids and Diet: ① Encourage children with a history of UTIs to drink adequate amounts of fluids. ② Avoid foods classified as drinks, caffeine, citrus, chocolate, and food colorants (10, 14).	Provide individualized advice on adequate fluid intake and avoidance of bladder-irritating drinks/foods where relevant.	5	B
Follow-up instructions	39. Follow-up Principles: Most pediatric patients have a favorable prognosis; however, recurrence is possible. Therefore, vigilance is essential, and regular follow-up appointments are necessary (14, 20).	Provide a written follow-up schedule and educate caregivers that good prognosis does not remove the need for vigilance for recurrence.	5	A
	40. Health Education: Healthcare professionals must clearly inform pediatric patients and their parents or caregivers (as appropriate) about the importance of timely treatment and completing the full course of therapy, key preventive measures, and long-term management requirements (14).	Before discharge, summarize treatment completion, warning signs, prevention measures, and long-term management requirements using a teach-back approach.	5	A
	41. Follow-up monitoring should include assessments of growth and development, urinalysis, complete blood count, and C-reactive protein levels. Urine cultures are indicated only if fever or symptoms of infection are present; routine monitoring is not required for asymptomatic individuals. Renal ultrasound should be repeated as clinically necessary (20, 21).	Clarify follow-up tests and avoid unnecessary routine cultures in asymptomatic children; advise culture when fever or infection symptoms recur.	2	B
	42. Imaging Follow-up: ① Renal-bladder ultrasound: Children with acute pyelonephritis and/or VUR should undergo follow-up imaging after 6 months. ② Radionuclide imaging: Routine testing is not required after the first urinary tract infection. However, all children with Grade IV-V VUR should undergo a renal cortical static scan using technetium-99 m dimercaptosuccinic acid (DMSA) at least 6 months after a febrile UTI to detect renal scarring (15, 21).	Explain imaging follow-up for pyelonephritis, VUR, or high-grade reflux and specify approximate timing to improve attendance.	5	B

## 4 Discussion

### 4.1 The importance and scientific validity of evidence summary on health education for pediatric UTIs

Pediatric UTI is a common condition with a high recurrence rate. Effective health education is therefore essential for prevention and home-based management. Accurate disease awareness, early symptom recognition, and proactive preventive measures are critical to reducing recurrence, improving patient outcomes, and enhancing family quality of life. Following a systematic search, 14 relevant articles were included. Through quality appraisal and evidence grading, 42 best-evidence statements across eight domains were synthesized. The overall quality of the evidence is high, indicating strong scientific rigor and considerable clinical utility.

### 4.2 The analysis of the best evidence summary on health education for pediatric UTI

#### 4.2.1 Disease awareness and recognition

Evidence statements 1–9 in this study focus on disease awareness and symptom recognition in pediatric urinary tract infection (UTI). The clinical manifestations of UTI in children are diverse. They range from asymptomatic bacteriuria and urinary irritative symptoms—with or without fever and gastrointestinal disturbances such as abdominal pain and vomiting—to potentially life-threatening sepsis. In infants and young children, symptoms are particularly nonspecific, complicating diagnosis relative to adults and often leading to delayed treatment. This delay increases the risk of serious complications, including renal scarring and end-stage renal disease. Almatrafi et al. (23) reported that most parents cannot recognize UTI symptoms in their children and lack knowledge of associated risks and treatment, contributing to misdiagnosis or poor treatment adherence. Similarly, Gates et al. (4) observed that parents frequently experience fear and anxiety regarding their child’s UTI, an emotional response rooted in insufficient understanding of the disease’s presentation. When children exhibit nonspecific symptoms, parents struggle to identify the underlying problem. Consequently, lower parental knowledge of UTIs is associated with greater disease uncertainty. Thus, identifying gaps in parental knowledge regarding pediatric UTIs and their prevention is critically important.

The occurrence of pediatric UTI is closely associated with multiple factors, including age, vesicoureteral reflux, phimosis, bladder and bowel dysfunction (BBD), inadequate fluid intake, habitual urine retention, allergic predisposition, reduced immunoglobulin levels, anemia, and malnutrition (24–26). Identifying these predisposing factors enables adherence to standardized hygiene practices and targeted behavioral interventions, which significantly reduce and help prevent UTIs (1). Caregivers require not only knowledge of the disease, its risk factors, and complications but also essential practical skills. These include identifying and eliminating UTI risk factors; reducing the risk of urethral contamination (e.g., preventing vulvar and preputial inflammation, promoting good hygiene practices, and changing underwear daily); clearing pathogens from the bladder (e.g., ensuring complete bladder emptying, maintaining adequate fluid intake, voiding regularly, and avoiding urine retention); and recognizing early symptoms to enable prompt response (27).

#### 4.2.2 Assessment and diagnosis

Diagnosing UTI in infants and young children remains challenging. Current evidence suggests that UTI should be considered in any child with fever of unknown origin without an identifiable cause (3, 18). Urine culture is the “gold standard” for UTI diagnosis (18), but it is accurate only when specimen collection is performed properly. The collection technique directly influences both the timeliness and accuracy of diagnosis (19). Prompt assessment and diagnosis are essential to prevent short-term complications (e.g., sepsis) as well as long-term sequelae, including renal scarring, chronic kidney disease, and hypertension (28). However, due to difficulties in collecting urine specimens from infants and young children—coupled with their atypical clinical presentations—delayed or missed diagnoses are common. Ensuring correct and timely urine specimen collection is therefore clinically important. Research indicates that insufficient feeding prior to collection reduces the success rate of urine collection; thus, adequate feeding should be provided beforehand (29). It is recommended that infants be breastfed or bottle-fed for no more than 20 min, with collection initiated 20–25 min after feeding (19), as infants typically empty their bladders 20–30 min post-feeding.

Nevertheless, in clinical practice, healthcare providers often lack awareness of assessing the infant’s feeding status, resulting in missed optimal collection windows. Consequently, before collecting urine specimens from infants and young children, providers should strengthen feeding-related education and administer appropriate feeding based on the child’s age and body weight (allowing extended feeding time for those who have difficulty feeding). During the urine sample collection and testing process, aseptic technique must be strictly adhered to, and unnecessary delays avoided. If non-invasive methods fail to yield a clean urine sample, an invasive collection method should be employed to ensure sample accuracy, thereby enabling timely and precise diagnosis.

#### 4.2.3 Imaging examinations

The primary role of imaging is to identify congenital anomalies of the kidney and urinary tract (CAKUT) affecting long-term renal outcomes. Renal and bladder ultrasound (RBUS), a non-invasive, radiation-free screening tool, can assess structural abnormalities such as hydronephrosis and renal dysplasia. According to NICE guidelines, RBUS should be performed in all UTI patients aged <6 months, whereas it may not be routinely required in older children who respond well to treatment (14). Although this restrictive strategy is cost-effective, it may miss a considerable proportion of urinary tract abnormalities (21). As a first-line screening tool, guidelines generally recommend performing RBUS 2–4 weeks after the first febrile UTI. This timing avoids false-positive findings during the acute phase and enables better detection of renal and urinary tract abnormalities. For complicated or atypical UTIs—such as those presenting with sepsis, fever lasting >72 h, or non-*Escherichia coli* infection—RBUS should be performed during the acute phase (21).

Voiding cystourethrography (VCUG) is the gold standard for diagnosing and grading vesicoureteral reflux (VUR) (21) and can clearly delineate urethral and bladder anatomy, making it essential prior to planning surgical intervention for VUR (17). However, this technique has disadvantages, including radiation exposure and patient discomfort from catheterization. Most guidelines consider VCUG not a routine indication but rather reserved for cases where RBUS suggests urinary tract malformations or other specific clinical circumstances (30).

In contrast, the 2021 EAU/ESPU update suggests that relying solely on RBUS may miss up to 33% of children with structural abnormalities. Accordingly, it recommends that all children <1 year of age with febrile UTI undergo imaging to rule out VUR. The guidelines propose two strategies: the “bottom-up” approach (VCUG first, followed by DMSA scan if positive) and the “top-down” approach (DMSA scan first, followed by VCUG if positive) (17). Each method has its advantages. Comparative effectiveness analyses based on the RIVUR/CUTIE database indicate that although the top-down approach is associated with a higher risk of UTI recurrence, it reduces the need for VCUG and RBUS (30). Synthesizing the available evidence, VCUG is recommended in the following situations: after the first febrile UTI when the pathogen is non-*E. coli*, or when RBUS reveals renal dysplasia, high-grade hydronephrosis, ureteral dilatation, urothelial thickening, or bladder abnormalities. VCUG is also indicated after every second febrile UTI episode. Furthermore, VCUG is always required prior to planning surgical intervention for VUR (21).

#### 4.2.4 Treatment and medication

Antibiotic treatment decisions for pediatric UTI require a delicate balance between acute infection control and long-term renal outcomes. Empirical antibiotic therapy should be initiated immediately after obtaining a urine culture specimen (15, 20). Its core value extends beyond eradicating infection and improving clinical symptoms to include preventing bacteremia and renal parenchymal damage, particularly in neonates during the first few months of life when the immune system is immature (20). Regarding treatment duration, guidelines provide stratified recommendations based on infection site and age. Febrile uncomplicated UTI warrants 7–10 days of treatment, whereas complicated UTI requires 10–14 days. For children >3 months of age with lower urinary tract infection confined to the lower tract, the duration may be shortened to 5 days. For infants <3 months of age—owing to their immature immune system, incomplete blood–brain barrier, and high risk of complications—intravenous administration for 10–14 days is recommended to ensure adequate renal tissue penetration and complete eradication of infection (15, 18, 20, 21). However, the long-term benefits of prophylactic antibiotics remain controversial. Although they reduce UTI recurrence rates, their efficacy in preventing renal scarring is limited (31). Studies have shown no significant difference in the incidence of renal scarring between prophylaxis and control groups. Moreover, long-term use may increase the risk of recurrence by 2.4-fold (16). Consequently, guidelines have shifted away from routine prophylactic antibiotic use in children with normal urinary tract anatomy and no bladder and bowel dysfunction. Instead, prophylaxis should be considered on an individualized basis only for those with moderate to severe (grade III–V) vesicoureteral reflux or recurrent febrile infections with risk of renal injury (16).

Adherence is a critical yet often overlooked determinant of treatment success. Studies indicate that among children with recurrent UTI, only 32.2% adhere to long-term antimicrobial regimens, and 19% completely fail to use the prescribed medication (15). This gap stems from several factors: parental misconceptions that treatment can be discontinued once symptoms improve, difficulties in administering medication to children, fatigue with long-term therapy, and excessive concerns about antibiotic safety. In summary, current evidence does not support routine prophylactic antibiotic use in children with recurrent UTI following their first infection. Treatment success depends not only on the prescription itself but also on improving adherence through parental education—covering disease risk factors, the importance of completing full treatment courses, and the benefits and barriers of preventive behaviors—alongside systematic strategies such as simplified dosing regimens and digital reminder tools. Only by achieving a delicate balance among efficacy, safety, cost-effectiveness, and antimicrobial resistance control can long-term renal outcomes in children be maximally protected.

#### 4.2.5 Recurrence prevention

Children, as a special population with limited self-protection capabilities, remain susceptible to various adverse factors that may lead to infection. The prevention of pediatric urinary tract infections (UTIs) largely depends on home-based management. Therefore, addressing modifiable risk factors is essential to prevent recurrent infections.

#### 4.2.6 Bladder and bowel dysfunction

Studies have identified BBD as a significant risk factor for both the occurrence and recurrence of pediatric UTIs, as well as a key contributor to the progression of renal scarring (15, 32). BBD refers to lower urinary tract dysfunction, clinically characterized by a range of lower urinary tract and bowel symptoms, including urgency, urinary withholding, daytime urinary incontinence, constipation, and painful defecation (33). A major concern for the long-term prognosis of patients with BBD is incomplete bladder emptying, elevated intravesical pressure, and invasion of the urothelium by enteric pathogens—all of which contribute to UTI recurrence (34). Furthermore, fecal retention due to constipation can lead to urinary stasis by compressing the bladder and elongating the urethra, thereby promoting pathogen adhesion and increasing UTI risk (35).

Conservative treatment for BBD includes timed voiding, pelvic floor awareness training, fluid management, and treatment or prevention of constipation. Approximately half of patients experience improvement with conservative measures alone (33). Urotherapy, constipation management, and other conservative strategies are central to BBD treatment. Studies indicate that adding secondary non-pharmacological interventions to urotherapy and constipation management can improve lower urinary tract symptoms and reduce the risk of UTI recurrence, with far fewer side effects than pharmacological or surgical treatments (34).

For children with UTI and inadequate fluid intake, increased fluid consumption is encouraged. Adequate hydration reduces bladder retention time and promotes bacterial clearance. However, fluid overload may lead to bladder overdistension, which can induce voiding dysfunction and increase post-void residual (PVR) volume. A voiding frequency/fluid intake chart can assist parents in monitoring their child’s voiding frequency and fluid intake (15). Timed voiding is recommended, typically every 2–3 h during the day. Older children may use watch alarms or timers as reminders to reduce urine retention time and avoid bladder overdistension (12). Additionally, some children habitually delay voiding due to concerns about insufficient privacy or poor hygiene in school bathrooms. Behavioral issues arising during toilet training—such as habitual resistance, fear or anxiety, and power struggles with parents or caregivers—may also contribute to voiding delay. The goal of corrective voiding interventions is to restore normal bladder and sphincter function and reestablish healthy voiding habits. A daily record should be maintained after initiating such interventions. A voiding diary should include the time and volume of each void, timing of incontinence episodes, fluid intake, bowel movement times, and episodes of fecal incontinence. Parents and age-appropriate children should be educated on normal voiding habits. Key recommendations include monitoring proper voiding posture (avoiding positions that hinder voiding, such as forcefully crossing the legs, squatting, or applying perineal pressure with hands or heels), encouraging voiding proactively before the urge is felt, emptying the bladder completely, and avoiding abdominal straining. A reward system is also recommended to enhance children’s adherence and self-confidence (12).

#### 4.2.7 Nutrition and immunity

Regarding dietary nutrition, Uwaezuoke et al. identified malnutrition as a predisposing factor for pediatric UTI (22). Secretory immunoglobulin A (sIgA) in urine represents a defense mechanism against UTIs, and its role in the pathogenesis of UTI episodes has been reported. Low urinary sIgA may be an important predisposing factor for recurrent UTIs. One of the impacts of malnutrition on the immune system is a diminished IgA response (22). Therefore, children require adequate nutrition to enhance immunity and resist bacterial invasion. Caregivers should consistently monitor the child’s nutritional status to prevent malnutrition. Additionally, reducing intake of foods that may provoke bladder overactivity—such as caffeine, orange juice, tomato products, and spicy foods—is recommended (10).

Furthermore, a study by Gan et al. (36) found that vitamin D levels, particularly when below 20 ng/mL, are also a risk factor for UTIs. Gao et al. (37) reported that vitamin D supplementation reduced the overall risk of UTIs by 3%. Vitamin D exerts its protective effect by suppressing interferon-gamma production in the immune system and upregulating antimicrobial peptides in the bladder during *Escherichia coli* infection, thereby reducing urinary tract injury and subsequent inflammation (36). Consequently, children should be encouraged to engage in outdoor activities and increase sunlight exposure to promote vitamin D synthesis, with supplementation provided when necessary to enhance immune defense.

#### 4.2.8 Hygiene practices

Poor perineal hygiene increases UTI risk. Parents, caregivers, and growing boys should be guided on proper care and hygiene of the uncircumcised penis. During bathing, the penis should be cleaned with water or a non-irritating, age-appropriate soap, without forcibly retracting the foreskin to avoid inducing or exacerbating urethritis (12). In addition, diapers should be changed frequently to prevent diaper dermatitis and skin irritation.

#### 4.2.9 Follow-up management

Most children with UTI have a favorable prognosis. However, those with coexisting urinary tract malformations or functional abnormalities are prone to recurrence and require regular follow-up. Follow-up management is a critical ongoing process following discharge, and caregivers should strictly adhere to prescribed medication regimens. Clinical symptoms typically improve within 1–2 days after initiating antibiotic therapy. Nevertheless, completing the full treatment course and attending regular outpatient follow-up visits remain essential—even after symptom resolution—to facilitate timely detection and management of potential recurrence. For specific clinical situations associated with increased recurrence risk, the following recommendations are warranted: (1) In children with recurrent febrile UTI, evaluation for bladder and bowel dysfunction (BBD) is advised. (2) If the causative pathogen of the first febrile UTI is non-*Escherichia coli*, an assessment for vesicoureteral reflux (VUR) should be performed. (3) For children over 1 year of age with febrile UTI caused by *E. coli*, VUR evaluation is recommended after the second episode (17). Furthermore, in children with high-grade VUR and reflux nephropathy, monitoring for renal scarring and long-term renal outcomes should be conducted at least 6 months after a febrile UTI episode. Long-term, regular follow-up of growth and development, blood pressure, proteinuria, and renal function is essential to enable early identification and management of potential long-term complications (15, 20, 21, 38).

## 5 Limitations

This evidence summary has several limitations. First, only 14 of 1,285 initial records met the inclusion criteria, which may limit the generalizability of the findings. Second, among the five guidelines assessed with AGREE II, only two achieved Grade A, and the “Applicability” domain scored relatively low across several guidelines. Third, no randomized controlled trials were included, limiting causal conclusions about specific educational strategies. Fourth, the search was limited to Chinese and English publications, and most sources originate from Europe, Asia, and North America, which may limit applicability to other settings. Fifth, direct input from patients or caregivers was not systematically integrated. Finally, given the dynamic nature of clinical guidelines, some evidence items may require updating.

## 6 Conclusion

This study summarized evidence on comprehensive health education for children with urinary tract infection (UTI) across the full disease continuum. Based on the distinct physiological characteristics of affected children and the knowledge needs of their caregivers, this evidence summary provides scientific and systematic health education guidance across eight aspects: disease awareness and symptom recognition; diagnosis, assessment, and urine specimen collection; imaging examinations; treatment and medication; relapse prevention; and follow-up management. These findings can support clinical and community healthcare professionals in developing and implementing evidence-based health education interventions and practical protocols for pediatric UTI. Translating this best evidence into clinical practice will ultimately improve health outcomes and quality of life for children with UTI. Given that some evidence is derived from international studies, future health education interventions should be culturally adapted to local contexts, taking into account the child’s individual situation, family needs, and available medical resources. Optimal evidence should be selectively applied based on specific clinical settings and caregiver requirements.

## Data Availability

The original contributions presented in the study are included in the article/supplementary material, further inquiries can be directed to the corresponding author.
